# Determination of Risk Factors for Venous Thromboembolism by an Adapted Caprini Scoring System in Surgical Patients

**DOI:** 10.3390/jpm9030036

**Published:** 2019-07-17

**Authors:** Bui My Hanh, Le Quang Cuong, Nguyen Truong Son, Duong Tuan Duc, Tran Tien Hung, Duong Duc Hung, Tran Binh Giang, Nguyen Hoang Hiep, Hoang Thi Hong Xuyen, Nguyen Thi Nga, Dinh-Toi Chu

**Affiliations:** 1 Tuberculosis and Lung Disease Department, Hanoi Medical University, Hanoi 100000, Vietnam; 2 Department of Neurology, Hanoi Medical University, Hanoi 100000, Vietnam; 3 Director Board, Cho Ray Hospital, Ho Chi Minh City 700000, Vietnam; 4 Center for Health Insurance and Multilateral Payment in The Northern Region, Viet Nam Social Security, Hanoi 100000, Vietnam; 5 Department of General Administration, Bach Mai Hospital, Hanoi 100000, Vietnam; 6 Director Board, Viet Duc Hospital, Hanoi 100000, Vietnam; 7 Institute for Research and Development, Duy Tan University, 03 QuangTrung, Danang 550000, Vietnam; 8 Faculty of Biology, Hanoi National University of Education, Hanoi Vietnam 100000, Vietnam; 9 School of Odonto Stomatology, Hanoi Medical University, Hanoi Vietnam 100000, Vietnam

**Keywords:** venous thromboembolism, Caprini score, risk assessment model, risk stratification, thromboembolism prophylaxis

## Abstract

Venous thromboembolism (VTE) is a frequent preventable complication among surgical patients. Precise risk assessment is a necessary step for providing appropriate thromboprophylaxis and reducing mortality as well as morbidity caused by VTE. We carried out this work to define the rate of VTE postoperatively, following a Caprini score, and to determine VTE risk factors through a modified Caprini risk scoring system. This multicenter, observational, cohort study involved 2,790,027 patients who underwent surgery in four Vietnamese hospitals from 01/2017 to 12/2018. All patients who were evaluated before surgery by using a Caprini risk assessment model (RAM) and monitored within 90 days after surgery. The endpoint of the study was ultrasound-confirmed VTE. Our data showed that the 90-day postoperative VTE was found in 3068 patients. Most of VTE (46.97%) cases were found in the highest risk group (Caprini score > 5). A total of 37.19% were observed in the high risk group, while the rest (15.84%) were from low to moderate risk groups. The likelihood of occurring VTE was heightened 2.83 times for patients with a Caprini score of 3–4, 4.83 times for a Caprini score of 5–6, 8.84 times for a score of 7–8, and 11.42 times for a score of >8, comparing to ones with a score of 0 to 2 (all *p* values < 0.05). Thus, the frequency of postoperative VTE rises substantially, according to the advanced Caprini score. Further categorizing patients among the highest risk group need delivering more appropriate thromboprophylaxis.

## 1. Introduction

Venous thromboembolism (VTE) (e.g., deep vein thrombosis (DVT) or pulmonary embolism (PE)) is a prevalent complication, which may lead to morbidity and mortality (MAM) in patients of both general medical and surgical conditions. The affliction regarding the disease is significant, as the annual rate of VTE in the Caucasian population is about 1 to 2 per 1000 patients each year [1,2]. Until now, research on the frequency of VTE is still deficient in the Asian population, probably because of the previous impression that VTE was not so frequent in Asia. Nevertheless, an increased number of publications have reported a clinical significance of VTE in the respective Asian population over recent years, although the reported rate has generally been lower than the rate among Western countries [3,4,5]. 

The inadequacy of an appropriate risk measure, limited data, atypical clinical symptoms of VTE leading to misdiagnosis, and the lack of postoperative follow ups are some restrictions encountered in evaluating the true nature of the disease and measurement of its complications. There are several surgery-related risk factors for VTE, including infection, immobilization, and dehydration in response to the specific surgical type and duration. Patients with malignancy, advancing age, obesity, or varicose veins are likely to obtain a higher risk of VTE. Recently, the extended application of prophylaxis, early mobilization, and promoted perioperative care have minimized the risk of VTE in patients following surgical treatment. Therefore, accurate and timely assessment of risk factors for thromboprophylaxis is needed to limit VTE occurrence and associated complications [6,7]. 

In order to determine VTE risks in surgery patients, several researchers recommended the use of individual patient risk assessments. Numerous risk scoring systems that estimate and classify patients following their risks have been proposed to predict VTE risks, in which the most outstanding cases are those established by Caprini, Kucher, Cohen, and NICE guidelines [8,9,10,11]. These models evaluate the likelihood of VTE occurrence based on exposure-related risk factors (medical comorbidities or surgical types) and predisposing characteristics (clinical and biological factors) attached to a specific risk factor score. A cumulative score is then generated by totalizing these scores in order to stratify VTE risk levels and provide appropriate recommendations for prophylaxis. 

The Caprini RAM facilitates the derivation of VTE risk based on a consolidation of published data with clinical experience. Because it is categorical and relatively easy to estimate, the Caprini RAM has been adopted worldwide. This model has been modified and validated in foreign studies. Particularly in China, the validation of this RAM has been applied to studies conducted on hospitalized patients for assessing VTE risk [12]. Nevertheless, to date, there is still limitation of studies validating the Caprini risk scoring system in the Vietnamese population. Therefore, we conducted this work to estimate the frequency of postoperative VTE and evaluate the reliability and validity of the modified Caprini scoring method in classifying risks for thromboprophylaxis. 

## 2. Materials and Methods

### 2.1. Study Design

A multicenter, observational, cohort study was implemented at four participating hospitals, including Hanoi Medical University Hospital, Cho Ray Hospital, Viet Duc Hospital, and Bach Mai Hospital in Vietnam from January 2017 to December 2018.

### 2.2. Patients

All adult patients (≥18 years old) who experienced either elective or emergency surgeries in participating hospitals were recruited into the study. Patients were excluded from the study if they underwent any of conditions as follows: (1) Diagnosis of VTE at the time of hospital admission; (2) any anticoagulant treatment during the admission; (3) pregnancy; (4) contraindication to therapy of anticoagulant for any reason; or (5) receiving antiplatelet drug.

### 2.3. Adapted Caprini Risk Scoring System

The Caprini risk assessment score was utilized in order to strengthen the observance of VTE prophylactic regimens for patients in both medical and surgical conditions. Each separated risk factor was assigned to specified scores ranking 1–5 points, depending on their influence on the likelihood of thrombosis. The individualized risk factors were calculated and then totalized to generate an accumulative risk score in order to distribute patients to four risk levels, including “low risk” (0–1 points), “moderate risk” (2 points), “high risk” (3–4 points), and “highest risk” (≥5 points) [9]. The RAM was adjusted to contain the clinical criterion only. Laboratory test measurements, such as Factor V Leiden, prothrombin 20110A, serum homocysteine, anticardiolipin antibodies, and lupus anticoagulant, were not included in the study.

### 2.4. Study Procedure

Clinical data were obtained via a standardized process at each hospital by a physician-led team. Risk factors for individuals were considered and then totalized to identify cumulative VTE risk and related levels of risk.

Patients were suspected as having DVT when symptoms such as swelling, pain, and tenderness in one or two legs (most commonly in the calf), heavy soreness when bending legs, feeling of warmth around swollen area, or red or discolored skin were discovered. Cases were suspected as having pulmonary embolism with symptoms such as unexplained breathlessness, chest pain when breathing in, or even sudden collapse. 

The primary outcome was a clinically suspected, ultrasound-confirmed VTE after surgery, consisting of upper or lower limb DVT and PE. DVT diagnosis was confirmed using Dupplex ultrasonography or venography, while detection of PE was performed using pulmonary angiography, a computed tomography (CT) scan, magnetic resonance imaging (MRI), or a ventilation-perfusion (V/Q) scan. The clinical symptoms and signs of VTE were assessed every 3–5 days during the postoperative period in patients who remained in hospital for inpatient care.

For patients who were discharged from hospital, they were re-examined or called for interview by phone on a specified day to detect VTE-suspected cases within 90 days after surgery. After being discharged from hospital, patients were instructed to report to a health worker if any such symptoms occurred at home. At the time of discharge, the score was renovated by treating the physician with any additional risk factor variables. Patients were classified in accordance with the risk scores, and the rate of postoperative VTE was estimated following the Caprini score in order to appraise the efficacy of the adapted risk scoring system. 

### 2.5. Ethics Approval and Consent to Participate

All procedures performed in studies involving human participants were in accordance with the ethical standards of the institutional and/or national research committee and with the 1964 Helsinki declaration and its later amendments or comparable ethical standards. This research was accepted by the Ethical Review Board of Hanoi Medical University (Approval No. IRB 0003121). Informed Consent is not applicable.

### 2.6. Statistical Analysis

The distribution of the Caprini score, the frequency of VTE by the risk score category, and the odd ratios for VTE by comparing the Caprini score categories to each other were calculated using a Chi-square test. The relative risks for each risk factor and the 95% confident interval were reported using a Chi-square test. The results were regarded as a statistical significance if *p* value < 0.05. All analyses were conducted using STATA 12.0 and SPSS 23.0.

## 3. Results

A total of 2,790,027 patients who experienced either elective or emergency surgeries were enrolled in our study. The gender distribution showed 50.1% males and 49.9% females. The 41–60 age group (35.15%) accounted for the highest proportion, followed by 32.7% patients over 61 years of age. 

**Figure 1:**
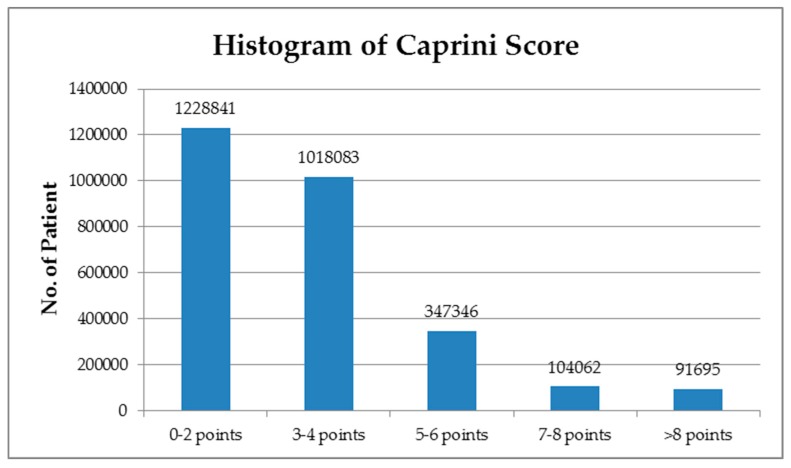
Histogram of the adapted Caprini scores.

**Figure 2:**
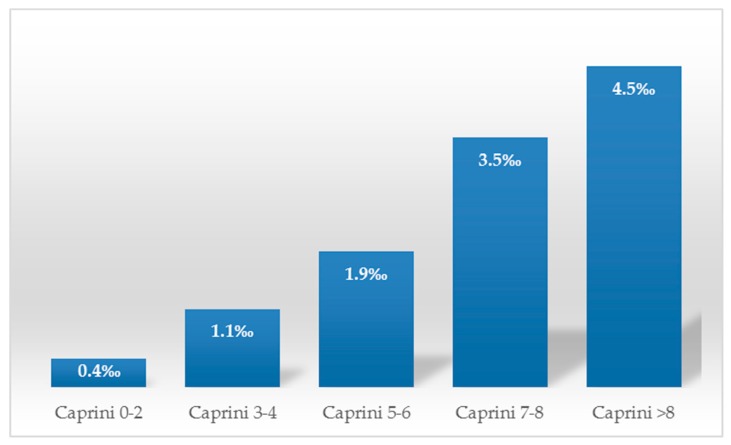
Rates of venous thromboembolism (VTE) after surgery in each group of Caprini scores.

In terms of the Caprini score, patients were placed into five subgroups: 0–2 points (44.04%), 3–4 points (36.5%), 5–6 points (12.45%), 7–8 points (3.7%), and >8 points (3.3%) (Figure 1). A total 3068 cases of 90-day postoperative VTE were identified using ultrasonographic screening. Of these, the majority of VTE cases (46.97%) was observed in the highest risk group; 37.19% were derived from high risk group; and the rest of the 486 VTE patients (15.84%) were from the group of low and moderate risk (Table 1, Figure 2). 

Patients among the >8 score group had significantly greater likelihood of developing postoperative VTE in comparison with the group of 3–4 points (odds ratio (OR) = 4.04; 3.61–4.52, *p* < 0.001), the group of 5–6 points (2.37; 2.10–2.68, *p* < 0.001), or the group of 7–8 points (1.29; 1.12–1.49, *p* = 0.004). 

Furthermore, the risk of VTE occurrence increased in the 7–8 score group in comparison with the 3–4 score group (3.13; 2.78–3.52, *p* < 0.001) or the 5–6 score group (1.84; 1.61–2.09, *p* < 0.001) (Table 2). VTE risk factors in the Caprini risk scoring system were evaluated, and a relative risk (RR) was estimated. Our study identified older ages, acute myocardial infarction (MI) I (4.51; 3.34–6.09), heart and respiratory failure (4.58; 4.05–5.18 and 4.01; 2.71–5.94), lung disease (3.86; 3.33–4.49), acute renal failure (5.51; 4.82–6.30), hypertension (4.41; 4.11–4.73), immobility (2.18; 1.34–3.57), cancer (1.42; 1.24–1.64), varicose veins (50.26; 46.46–54.36), and peripheral vascular disease (21.43; 18.62–24.66) as significantly associated with postoperative VTE, while malignancy and chronic obstructive pulmonary disease (COPD) were statistically insignificant (*p* > 0.05). All of the parameters were found to have an advanced risk for the occurrence of VTE after surgery (Table 3). 

## 4. Discussion

Although venous thromboembolism remained a common postoperative complication, with numerous risk factors being conducive to the occurrence of the disease, VTE prophylaxis was shown to be underused, which is mainly because of limited awareness among the health care setting relating to the determination of high-risk patients who are in need of thromboprophylaxis [13]. The appearance of the risk assessment model is necessary in improving this current situation. The goal of our study is to verify the adapted Caprini risk scoring method, which is applied in stratifying the VTE risk in surgical patients on the basis of individualized risk factors. Our study showed that a higher risk of VTE was associated with the advanced Caprini score and the cumulative risk level. The rate of postoperative VTE was revealed to be approximately 0.04% in the low and moderate risk groups (0–2 points), 0.11% in the high-risk group (3–4 points), and 0.27% in the highest risk group (≥5 points). These results were lower than the findings reported in a study conducted by Bahl et al., which suggested the incidence rate of VTE to be 0.7%, 0.97%, and 1.94% in the low-moderate risk, high-risk, and highest risk groups, respectively [14]. 

According to the original Caprini risk assessment model, the highest risk group includes only patients with total score of five or more. A previous study suggested a marked growth in incidence of VTE in the Caprini score ≥5 patients, which ranges from 4% to 18% [15]. In the present study, we modified the Caprini RAM and divided the highest risk group into three separate subgroups (Caprini score of 5–6, 7–8, and >8) and recommended an extended duration of prophylaxis for each. The present study indicated that the rate of VTE went up from 0.19% to 0.45% with the total Caprini scores increasing from 5 to >8. Similarly, Pannucci et al. analyzed data of patients in 2016 who had received plastic surgery and thromboprophylaxis and observed an overall occurrence of DVT from 1.2% to 4.1% in accordance with the overall Caprini score in the highest risk patients (score ≥5) [16]. In an analysis of 704 otolaryngology surgical inpatients receiving chemoprophylaxis, the frequency of DVT rose gradually from 0 to 13.6% for patients with a score ranging from 5 to >8 [17]. The findings suggest that the incidence of a 90-day postoperative VTE seemed to have an upward tendency in the highest risk group as a cumulative risk score went up, which might be helpful for discovering patients who would earn the most benefit from prolonged thromboprophylaxis; however, the association between the duration of patients who continue to be at risk and the cumulative risk score needs more analysis. 

Our study presented that the acceleration in odds ratio (OR) for the acquired VTE was marked in cases with an advanced Caprini score in the highest risk group. Compared to the high risk group, the Caprini score of 5–6 cases presented a 1.7 times (95% CI; 1.55–1.88, *p* <0.001) possibility of occurring VTE after surgery, and the 7–8 score group showed a 3.13 times (95% CI 2.78–3.52, *p* < 0.001) advanced risk of postoperative VTE. The >8 score group demonstrated a 4.04 times (95% CI 3.61–4.52, *p* < 0.001) greater risk of postoperative VTE. Our results were lower than the findings reported by Kanchan et al. [18]. Bahl et al. conducted research on the validity of the adapted Caprini RAM in 8216 surgical patients and suggested that patients within the highest risk group were correlated with the higher likelihood of developing VTE after surgery (OR = 1.9; 1.3–2.6, *p* < 0.001) than the Caprini score of the 3–4 group [14]. Another study validating the risk scoring system in plastic surgical patients with VTE, conducted by Pannucci et al. [16], also demonstrated comparable results. The Caprini score groups of 7–8 and >8 have advanced risk of acquiring VTE after surgery, with OR = 4.5 and OR = 20.9, respectively, in comparison with the 3–4 score patients [16]. Our results indicated that further categorizing patients in the highest risk group is essential in determining the extent of risk more precisely in order to administer appropriate VTE prophylaxis, which is also consistent with the declaration of Cassidy et al. [19].

Regarding to risk factors of venous thromboembolism, it was found that increased age, acute myocardial infarction, varicose vein, cancer, heart failure, stomach ulcer, acute renal failure, hypertension, immobilizing >3 days, peripheral vascular disease, and a history of thrombosis increased the likelihood of occurring VTE. Of these, varicose veins and peripheral vascular disease were associated with the highest risk of acquiring VTE after surgery. These findings were well accepted by the literature [11,20,21,22]. Anderson et al. found that heart failure, respiratory failure, previous VTE, and prolonged immobility present supplemental and significant risk, with OR results ranging from 2 to 9 [20]. A systematic review of medical literature indicated thromboembolic history, increasing age, cancer, and varicose veins as independent risk factors for the occurrence of VTE [23]. 

However, malignancy and chronic obstructive pulmonary disease (COPD), which are identified influences on the occurrence of thromboembolic disease, were not perceived as significant in predicting VTE outcomes. Our result is somewhat contrasted to foreign studies. In a recent study on the status of thromboprophylaxis in a surgical patient, Petralia t al. showed various risk factors of VTE and emphasized patient-related risk factors, such as increasing age, malignancy, and previous VTE [24]. On the contrary, Clayton et al. suggested that the percentage of confirmed-DVT malignancy patients was much greater (20%) compared to non-DVT patients with malignancy (1%), however, the difference did not achieve statistical significance, which was confirmed by stepwise logistic regression (*p* > 0.05) [25]. 

## 5. Conclusions

It is necessary to enhance perception between health care settings relating to identification and intervention of venous thromboembolism. The incremental rise and thrombosis rate with an increasing risk score is of major importance. We suggest that modification of the Caprini scores to analyze the additional subgroup used in this paper will help clinical doctors to improve the current thrombosis prophylaxis protocol more accurately. This means that those patients with the highest score may have more intense prophylaxis, and any bleeding that is associated with this may be justified on the basis of high thrombotic risk.

## Figures and Tables

**Table 1:** Incidence of VTE within 90 days postoperatively.

	No. of Patients with VTE (%)	Relative Risk	*p* Value
Caprini 0–2	486 (0.04%)	_	_
Caprini 3–4	1141 (0.11%)	2.83 (2.55–3.15)	<0.001
Caprini 5–6	663 (0.19%)	4.83 (4.29–5.42)	<0.001
Caprini 7–8	364 (0.35%)	8.84 (7.72–10.13)	<0.001
Caprini >8	414 (0.45%)	11.42 (10.02–13.01)	<0.001

Note: *p* values were calculated using the Chi-square test, comparing patients with a score of 3 or more with the 0–2 score group.

**Table 2:** Odds ratio of postoperative VTE according to the Caprini score.

Caprini Score	5–6 Points	7–8 Points	>8 Points
3–4 points	1.7 (1.55–1.88) *p* < 0.0001	3.13 (2.78–3.52) *p* < 0.0001	4.04 (3.61–4.52) *p* < 0.0001
5–6 points	-	1.84 (1.61–2.09) *p* < 0.0001	2.37 (2.10–2.68) *p* < 0.0001
7–8 points	-	-	1.29 (1.12–1.48) *p* = 0.0004

Note: *p* values were calculated using the Chi-square test. Odd ratios were calculated by comparing Caprini score groups to each other.

**Table 3:** Individual risk factors for the occurrence of postoperative VTE.

Risk Factors	RR (95% CI)	Number of Patients	Number of Patients with Postoperative VTE	*p*
Age 41–60	2.71 (2.40–3.06)	980,708	1042	<0.001
Age 61–74	4.34 (3.84–4.89)	617,609	1050	<0.001
Age >74	5.37 (4.71–6.12)	296,650	625	<0.001
Acute MI	4.51 (3.34–6.09)	8759	43	<0.001
COPD	1.03 (0.61–1.75)	12,304	14	0.898
Heart failure	4.58 (4.05–5.18)	59,169	277	<0.001
Cancer	1.39 (1.21–1.61)	135,001	207	<0.001
Varicose vein	50.26 (46.46–54.36)	20,338	827	<0.001
Liver disease	2.29 (1.98–2.64)	80,812	196	<0.001
Stomach ulcer	2.62 (2.44–2.82)	502,953	1122	<0.001
Respiratory failure	4.01 (2.71–5.94)	5703	25	<0.001
Lung disease	3.86 (3.33–4.49)	44,816	182	<0.001
Acute renal failure	5.51 (4.82–6.30)	40,814	232	<0.001
Hypertension	4.41 (4.11–4.73)	550,498	1596	<0.001
Immobilizing >3 days	2.18 (1.34–3.57)	6685	16	0.0018
History of DVT/PE	5.81 (4.79–6.25)	4901	1170	<0.001
Peripheral vascular disease	21.43 (18.62–24.66)	9243	204	<0.001

Note: *p* values were calculated using the Chi-square test, comparing VTE and non-VTE patients for individualized risk factors; myocardial infarction, MI; chronic obstructive pulmonary disease, COPD; deep vein thrombosis, DVT; pulmonary embolism, PE.

## Data Availability

The material supporting the conclusion of this paper has been included within the article.

## Abbreviations

VTE	Venous thromboembolism
PE	Pulmonary embolism
CT	Computed tomography scan
MRI	Magnetic resonance imaging
RAM	Risk assessment model
V/Q	Ventilation-perfusion scan
